# The impact of severe rare chronic neurological disease in childhood on the quality of life of families—a study on MLD and PCH2

**DOI:** 10.1186/s13023-021-01828-y

**Published:** 2021-05-10

**Authors:** Louisa Ammann-Schnell, Samuel Groeschel, Christiane Kehrer, Saskia Frölich, Ingeborg Krägeloh-Mann

**Affiliations:** Department of Child Neurology, Children’s Hospital, University of Tübingen, Hoppe-Seyler-Str. 1, 72072 Tübingen, Germany

**Keywords:** Disease burden, Metachromatic leukodystrophy, Pontocerebellar hypoplasia type 2, Parents, Quality of life

## Abstract

**Background:**

Rare and severe neurological disorders in childhood not only heavily affect the life perspective of the patients, but also their caregivers and families. The aim of this study was to investigate the impact of such diseases on the family, especially on the quality of life and life perspectives of parents, but also on the families’ everyday life, based on the model of two diseases which have been well described in recent years with respect to symptoms and course: metachromatic leukodystrophy (MLD) and pontocerebellar hypoplasia type 2 (PCH2). PCH2 is a primary severe developmental disorder, while children with MLD initially develop normally and then progressively deteriorate.

**Methods:**

Using a semi-standardized questionnaire, 43 families with children suffering from MLD (n = 30) or PCH2 (n = 19) reported data on the severity of the illness/symptoms, on family support and the care situation, as well as on the circumstances of non-affected siblings and the parents’ work situation. In addition, the quality of life of parents and general family functioning was assessed using the PedsQL™ Family Impact Module [23]. Results for the latter were compared to published data from families with children without any chronic condition using student’s t-tests for independent samples. Potential factors influencing the PedsQL™ scores were analyzed using Spearman’s rank correlation.

**Results:**

Parents of children with MLD and PCH2 reported significantly lower health-related quality of life (HRQOL) compared to parents of healthy children (*P* < 0.001). Mothers showed significantly poorer HRQOL (*P* < 0.05) and were significantly more dissatisfied with their professional development (*P* < 0.05) than fathers, and this was seen in relation to their child's disease. Neither the form of disease (‘primary’ symptomatic PCH2 or ‘secondary’ symptomatic MLD), nor the severity of the child’s illness (in terms of gross motor and speech function) had a specific impact on HRQOL in families. However, the time from diagnosis and advanced symptoms in the terminal disease stage were experienced as especially distressing.

**Conclusions:**

This study illustrates that MLD and PCH2 affect mothers in particular, but also the entire family. This underlines the need for personalized care and counselling of parents and families, especially following diagnosis and during the end stage in a child with a severe, rare chronic neurological disorder.

## Background

Rare and severe neurological disorders in childhood not only heavily affect the life perspective of the patients, but also their caregivers and families [1–4].They are confronted with many different worries and challenges. Firstly, they realize that their child’s development is not as would be expected and the time to diagnosis may be lengthy and full of uncertainties, which cause anxiety [5]. In addition, being confronted with the diagnosis of a severe untreatable disease might cause even more distress to the parents [6]. Secondly, the recognition that the child’s condition is severe and chronic, requiring much support and care, completely changes everyday life, quality of life and the life perspective of the entire family [7, 8].

Pontocerebellar hypoplasia type 2 (PCH2) and metachromatic leukodystrophy (MLD) are two examples of rare and severe neurological disorders in childhood [8, 9]. PCH2 is a primary severe developmental disorder, while children with MLD initially develop normally and then progressively deteriorate [9, 10]. The different dynamics of the diseases, in addition to the different forms of severity (in MLD), may variably contribute to the burden associated with these diseases.

MLD is an autosomal recessive inherited neurodegenerative disorder with an estimated frequency of 0.6 in 100,000 live births of both genders in Germany [11]. A deficiency of arylsulfatase A (ASA) or, more rarely, of saposin B, a non-enzymatic activator protein, inhibits the enzymatic hydrolysis of sulfatide and results in progressive accumulation, primarily within lysosomes [12]. Sulfatides induce changes within the cells responsible for myelin maintenance, oligodendrocytes in the central nervous system and Schwann cells in the peripheral nervous system, which leads to demyelination and hence to severe neurological symptoms [13]. Based on age at onset, MLD can be classified into a late-infantile, a juvenile and an adult form [13, 14].

Children suffering from the late-infantile form initially develop normally and then, usually in the second year of life, develop symptoms such as gait problems, spasticity and ataxia [13, 15]. After an initial stage of relative stagnation, a stage of rapid regression follows in the third year, with loss of gross motor function, dysarthria, loss of speech and mental regression [14, 16, 17]. In most instances, death occurs about 5 years after the onset of clinical symptoms, but patients may remain in a near vegetative state for years [13, 14]. The disease course of the juvenile form is more variable, age at onset is defined as between 2½ and 16 years. Gait disturbance, concentration and behavioral problems and a decline in school performance characterize the first stage, which can last for several years [14, 16]. However, once independent walking is lost, gross motor function deteriorates as rapidly as in the late-infantile form, irrespective of age of onset [14]. The juvenile form leads to death many years after its onset [13, 18]. In the adult form, defined with an onset at the age of 16 years or later, the first stage is often characterized by behavioral abnormalities and even psychotic symptoms, followed or accompanied by a decline in intellectual capacity and motor function. Onset may be insidious and regression very slow [13]. At the time of the study, a therapeutic option was available for juvenile patients only, namely, stem cell transplantation in a presymptomatic or early symptomatic stage [18].

PCH, a group of very rare autosomal recessive inherited neurodegenerative disorders, are characterized by prenatal onset, a hallmark of which is an abnormally small cerebellum and ventral pons [19, 20]. The most frequently reported form, PCH type 2, is caused by mutations in the TSEN54 gene and is accompanied by progressive microcephaly, severe developmental delay and a severe dyskinetic or dyskinetic-spastic movement disorder. Feeding difficulties, gastroesophageal reflux, sleep disorders and apnea, as well as recurrent infections, are characteristic features of the disease, as is a severe epileptic seizure disorder, including frequent status epilepticus [20]. PCH treatment is only symptomatic and the prognosis is poor, as most patients do not reach puberty [10, 21].

As summarized above, the symptoms and course of disease for MLD and PCH2 have been systematically described in recent years. While PCH2 is associated with a primary severe developmental disorder, children with MLD initially develop normally and then progressively deteriorate. What both diseases have in common is that they are rare and severe childhood neurological disorders associated with a very high-care effort. Previous studies have shown that such severe life-limiting illnesses not only affect the patients themselves, but in many ways the whole family system [1–4], and that families cope with this challenge in different ways [6, 22].

*The aim of our study, therefore, was to investigate the impact of these diseases on the affected families, e.g. mothers, fathers and non-affected siblings*. This seems not only important for physicians, to improve prospective management, but also for the families as a basis for a better understanding of their circumstances as a first step in the direction of adjusting their life perspectives.

## Methods

### Recruitment

After approval by the ethics committee of the Faculty of Medicine Tübingen, in November and December 2016 parents of children suffering from MLD and PCH2 were asked to participate in this study. These families were known in our center within the context of systematic studies on the natural history of these diseases [8, 9] (recruited within the nationwide Leukonet natural history study, patient support groups in Germany [ELA Germany, MLD support group “Weisse Wolke” and PCH- Familie e.V.]). If interested, the parents were given the paper questionnaire along with an information letter to complete at home; both parents were asked to participate. Parents were included in the study after having signed the informed consent form.

### Survey design

Parents of children with MLD and PCH2 were surveyed based on a semi-standardized questionnaire. The questionnaire consisted of a general part and the Pediatric Quality of Life Inventory (PedsQL™) Version 2.0 Family Impact Module (FIM) [23]. The general part consisted of a mixture of quantitative and open questions. Quantitative questions included multiple choice questions and categorical scales, including validated scores like Viking Speech Scale (VSS) [24] and the Gross Motor Function Classification-MLD (GMFC-MLD) [16]. Further questions referred to the impact of the diagnosis, family support and care situation, as well as the situation of the non-affected siblings, the work situation of the parents and their satisfaction with their level of disease-specific knowledge. Rating scale questions with a scale of from 0 to 10 were used to metrically quantify opinions and attitudes on certain situations or topics. Open questions offered the possibility for parents to give information on aspects not covered by the standardized questions and were intended to support the quantitative analyses with exemplary quotes.

The influence of the child’s illness on the family’s quality of life was assessed in the PedsQL™ family impact module (FIM) [23], a standardized and established questionnaire module. The 36-item questionnaire measures parent-self-reported functioning due to the child’s illness in the following scales: physical health, emotional health, social functioning, cognitive functioning, communication and worry, as well as parent-self-reported family functioning based on daily activities and family relationships. Items are reversely scored and linearly transformed on a scale from 0 to 100 (0 = 100, 1 = 75, 2 = 50, 3 = 25, 4 = 0), so that higher scores indicate better functioning/less negative impact [23]. The Total Impact Score of the PedsQL™ FIM is composed of the sum of all 36 items divided by the number of items answered. The Parent Health-Related Quality of Life (HRQOL) Summary Score is composed of the sum of items from physical health, emotional health, social functioning, and cognitive functioning scales (20 items in total), divided by the number of items answered. The Family Functioning Summary Score is composed of the sum of items from the domains of daily activities and family relationships (8 items in total), divided by the number of items answered. Scale scores are calculated only if at least 50% of the items in the respective scale have been answered. For the PedsQL™ FIM Scales, Parent HRQOL and Family Functioning Summary Scores as well as the Total Impact Score, previous results found strong internal consistency scores [23]. The PedsQL™ FIM was used in the validated German translation [25]. Mothers and fathers were asked to complete this module separately and assess the impact of their child’s illness on their quality of life within a period of the past four weeks at the time of the survey. Results were compared to results from families with children without any chronic condition found in the literature using the same measure [26]. In this study, children were included in the group of children without any chronic condition whose parents denied that they suffered from nutritional problems, ADHD, asthma, bedwetting, constipation, depression, diabetes, food allergies, frequent headaches/migraines, recurrent abdominal pain, recurrent ear infections, or sleep problems. Among the participants, who were usually known by and approached to participate by undergraduate or graduate students, the majority (63.6%) reported being mothers [mean age 37.6 years (SD: 8.6)], having at least a bachelor’s degree and living in Wisconsin, United States [26].

Parents who needed help were contacted by telephone to help them fill in the questionnaires and ensure they understood the questions. Immediately after data collection, all pages of the questionnaire were anonymized with a code and kept separate from the data sheet on which personal information was requested.

### Data analysis

Descriptive analysis was performed to describe the characteristics of the study population. As the study was exploratory, all *P* values were being regarded as descriptive, *P* < 0.05 was considered statistically significant. The data was analyzed using IBM SPSS Statistics for Windows Version 24.0 (IBM Corp., Armonk, NY, USA). For smaller sample sizes, categorical variables and metrics where data was not assumed to be normally distributed, the median and range were calculated (for the age of the children, GMFC-MLD and VSS values, and the rating scale questions). All other continuous measurements were summarized as means ± standard deviations (SDs), and categorical measurements were summarized using frequencies with percentages.

To investigate the influence of the child’s illness on the family’s quality of life (Table 2), a t-test for independent samples was used to compare the difference in average scores of PedsQL™ FIM between the MLD and PCH2 sample, as well as its subgroups and the “no chronic condition” sample; however to compare the differences between each of the three disease forms, a Mann–Whitney-U test for independent samples was used because normal distribution could not be assumed due to small sample sizes.

In order to investigate the impact of time after diagnosis on the family’s quality of life a Mann–Whitney-U test was used to compare the difference in average scores of PedsQL™ FIM between the “less distressing” sample and the “more distressing” sample.

The influence of severity of symptoms on the family’s quality of life was investigated using Spearman’s rank correlation analysis in order to correlate the PedsQL™ FIM scores with potential drivers like GMFC-MLD, VSS values, perceptibility of visual stimuli, independent eating or presence of a PEG tube. A Mann–Whitney-U test was used to compare PedsQL™ FIM scores between families of more and less severely affected children, according to motor function/speech/vision/eating/presence of PEG tube (Table 3).

In addition, in order to investigate the influence of the child’s illness on mothers’ and fathers’ quality of life separately, a Mann–Whitney-U test was used to compare the average scores of PedsQL™ FIM between MLD and PCH2 mothers and between MLD and PCH2 fathers. And in order to compare the PedsQL™ FIM scores within families between MLD mothers and fathers and between PCH2 mothers and fathers a Wilcoxon signed-rank test for dependent samples was used.

## Results

### Study population

In total, data from 43 families (27 MLD, 16 PCH2) with 49 children (30 with MLD, 19 with PCH2) were analyzed in this study (Table 1). From the 30 children with MLD, 8 had the late-infantile and 21 the juvenile form. The only case of adult MLD (22.7 years at onset) was added to the juvenile MLD sample during analysis, because of the otherwise small sample size. Five MLD and four PCH2 families were included in the study that had two affected children each. In six of these cases, data were collected from both affected siblings. All other sibling children (overall 50 (half-) brothers and sisters) were not affected by MLD or PCH2. Seven MLD patients had received stem cell transplantation and one MLD patient enzyme replacement therapy. Of the 49 patients, 12 (6 with MLD, 6 with PCH2) had already died at the time of the survey. If this was the case, parents were asked to answer all questions according to their situation during the final phase of their child’s illness.

**Table 1:** Classification of disease type families and affected children

	Type of MLD				
	Late-infantile (< 2½ years at onset)	Juvenile (2½ to 16 years at onset)	Adult (> 16 years at onset)	PCH2	Total
Families (n)	8	18	1	16	43
Mothers (n)	8	16	1	16	41
single mothers (n)	1	3	0	1	5
Fathers (n)	7	14	1	13	35
Children (n)	8	21	1	19	49 (25♀/24♂)
age in yrs. at time of survey^a^	5.8 (3.1–8.4)	19.1 (3.2–41.4)	31.1 (31.1–31.1)	9.9 (0.8–20.0)	11.6 (0.8–41.4)

^**a**^Median age (range)

MLD, metachromatic leukodystrophy; PCH2, pontocerebellar hypoplasia type 2

### Influence of the child’s illness on the family’s quality of life

Table 2 gives means and standard deviations for the PedsQL™ FIM in comparison to results from families with children without any chronic condition [26].

**Table 2:** PedsQL™ Family Impact Module

Sample	Total Impact Score	Parent HRQOL	Family Functioning
MLD and PCH2 (n = 43/43/42)	57.3*** (17.5)	60.8*** (18.3)	49.8*** (22.2)
Late-infantile MLD (n = 8/8/8)	48.3*** (15,3)	54.8*** (20.8)	35.4*** (19.2)
Juvenile MLD (n = 19/19/18)	59.1*** (19.6)	61.1** (20.9)	56.2** (22.0)
PCH2 (n = 16/16/16)	59.7*** (15.5)	63.4* (15.2)	49.9*** (21.7)
MLD Mothers (n = 25/25/24)	53.1*** (18.8)	55.6*** (19.7)	47.8*** (24.3)
MLD Fathers (n = 22/22/22)	58.6*** (19.3)	63.0* (21.5)	51.9*** (21.5)
PCH2 Mothers (n = 16/16/16)	55.9*** (17.9)	58.6** (17.8)	47.4*** (23.6)
PCH2 Fathers (n = 13/13/13)	67.7 (16.1)	73.6 (16.6)	56.4* (24.3)
No chronic condition (n = 546) [26]	73.2 (13.6)	71.2 (14.9)	67.6 (18.4)

Standard deviation presented in parentheses. Different case numbers result from the fact that only sufficiently completed scales could be used. Higher values equal better functioning/less negative impact

PedsQL™, Pediatric Quality of Life Inventory [23]; MLD, metachromatic leukodystrophy; PCH2, pontocerebellar hypoplasia type 2; HRQOL, health-related quality of life

^*^P value is significant at < 0.05 level; **P value is significant at < 0.01 level; ***P value is significant at < 0.001 level. Comparison between individual samples and the “no chronic condition” sample

**Fig. 1:**
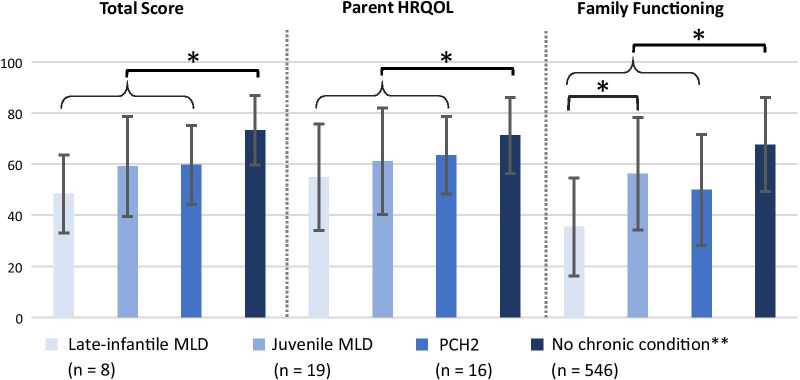
PedsQL™ Family Impact Module. Higher values equal better functioning/less negative impact. *P value is significant at < 0.05 level; **Medrano et al. [26]. Comparison between individual samples, as well as between individual samples and the ‘no chronic condition’ sample. PedsQL™, Pediatric Quality of Life Inventory [23]. MLD, metachromatic leukodystrophy. PCH2, pontocerebellar hypoplasia type 2. HRQOL, health-related quality of life

MLD and PCH2 families reported significantly poorer HRQOL than the families with healthy children in the overall domains of Total Impact Score, Parent HRQOL and Family Functioning Summary Scores. Furthermore, when divided into the three different forms of disease, these families reported significantly poorer HRQOL than the families with healthy children (Fig. 1). However, the comparison of the means for the families in the PedsQL™ FIM between each of the three disease forms showed no significant difference, except significantly poorer Family Functioning (*P* < 0.05) in the late-infantile MLD families compared to the juvenile MLD families.

When analyzed according to disease and gender of the parent, MLD mothers and fathers, as well as PCH2 mothers, exhibited significantly poorer HRQOL compared to the families with healthy children. Conversely, PCH2 fathers only exhibited a significantly poorer Family Functioning.

### Burden of diagnosis

#### Time to diagnosis

On average, time to diagnosis after the onset of first symptoms was 25.1 months (SD: 54.9, n = 43), by which time families had visited 2.9 (SD: 1.7, n = 45) different medical facilities. Mean time to diagnosis was 6.1 months (SD: 4.0) with a mean number of 3.8 visits to a doctor (SD: 2.1) in families with late-infantile MLD (n = 8), 41.4 months (SD: 78.3, n = 18) with a mean number of 2.4 visits to a doctor (SD: 1.2, n = 19) in families with juvenile MLD, and 16.7 months (SD: 28.9, n = 17) with a mean number of 3.1 visits to a doctor (SD: 1.9, n = 18) in families with PCH2. On a rating scale of 0 to 10, with 0 being “the time to diagnosis was not distressing” and 10 “the time to diagnosis was very distressing”, the parents of MLD children (n = 27) reported a median value of 9.3 (range 0.6–10.0) and the parents of PCH2 children (n = 16) a median value of 9.0 (range 0.6–10.0).

As an example, parents who reported the time to diagnosis as rather distressing commented,

“Despite the abnormalities, doctors were initially unable to find anything, which was very stressful for us. The diagnosis has at least finally given us an explanation.” (juvenile MLD, 42 months to diagnosis)

And “It was very, very distressing to see my child changing, in pain and no doctor/therapist being able to help.” (late-infantile MLD, 11 months to diagnosis)

However, other parents who did not find it distressing noted,

“At first, this period was not very stressful, because nobody thought of a severe illness. [But] from the day the blood was taken until we got the result, the uncertainty was very bad.” (late-infantile MLD, 5 months to diagnosis)

And “[This period was] not really [stressful], because you did not expect a bad diagnosis.” (juvenile MLD, 5 months to diagnosis)

#### Diagnosis

Parents answered that they had received the diagnosis personally in 85.7% of cases (n = 49), by post in 6.1% of cases, over the telephone in 4.1% of cases and 4.1% didn’t answer this question. A total of 95.2% of parents who received the diagnosis personally (n = 42) stated that this took place in a doctor’s room, 2.4% had received the diagnosis at the bedside and 2.4% in a corridor. When asked who informed them of the diagnosis, 91.8% stated “a doctor”, 2.1% stated “a secretary” and 6.1% didn’t answer this question.

On a rating scale of 0 to 10, with 0 being “I did not feel well informed about the illness right after the diagnosis” and 10 “I felt well informed about the illness right after the diagnosis”, the parents of MLD children (n = 27) reported a median value of 5.0 (range 0.0–10.0) and the parents of PCH2 children (n = 16) a median value of 2.4 (range 0.0–10.0). However, on a rating scale of 0 to 10, with 0 being “I was not satisfied with the way I was informed of the diagnosis” and 10 “I was very satisfied with the way I was informed of the diagnosis”, the parents of MLD children (n = 27) reported a median value of 8.5 (range 0.0–10.0) and the parents of PCH2 children (n = 16) a median value of 7.0 (range 0.0–10.0). Parents repeatedly reported the following as particularly helpful in this situation: honesty about the severity and a comprehensible explanation of the condition, as well as empathic handling. For example, one parent wrote,

“The professor spoke openly about the course of the disease, but very sensitively. Always there for questions.” (juvenile MLD)

The most disturbing factors reported were insensitive behavior towards the parents, ignorance of the disease on the part of the doctors, as well as the passing on of unconfirmed diagnoses. For example, one parent noted,

“The sentence: “Your child will die.” has burnt itself into my memory to this day. This sentence was said right at the beginning.” (late-infantile MLD)

#### Time after diagnosis

The median time between diagnosis and the time of this survey was 10 years (range 1–27 years). On a rating scale of 0 to 10, with 0 being “the time right after diagnosis was not distressing” and 10 “the time right after diagnosis was very distressing”, both the parents of MLD children (n = 27) and the parents of PCH2 children (n = 16) specified a median value of 10.0 (range 0.0–10.0). For example, parents who reported the time right after diagnosis as distressing commented,

“It felt as if our son had already died, as if the carpet had been pulled from under our feet.” (late-infantile MLD)

And “[It was] the most terrible time in my life, I saw no meaning in it anymore and actually just wanted to finish it.” (PCH2)

However, two of the few parents who did not find it distressing (see range 0.0–10.0) noted,

“(…) We were in high spirits because we knew that we could use prenatal diagnostics for the second child. We were relieved that it was PCH 2 and not an unknown form, on which far fewer publications are available. After clinical diagnosis: one could finally search specifically and read studies and find other parents and children.” (PCH2)

And “[We felt] relief, especially because it was clear that [name of the child] has recessively inherited PCH 2 and no one is at fault for his illness.” (PCH2)

**Fig. 2:**
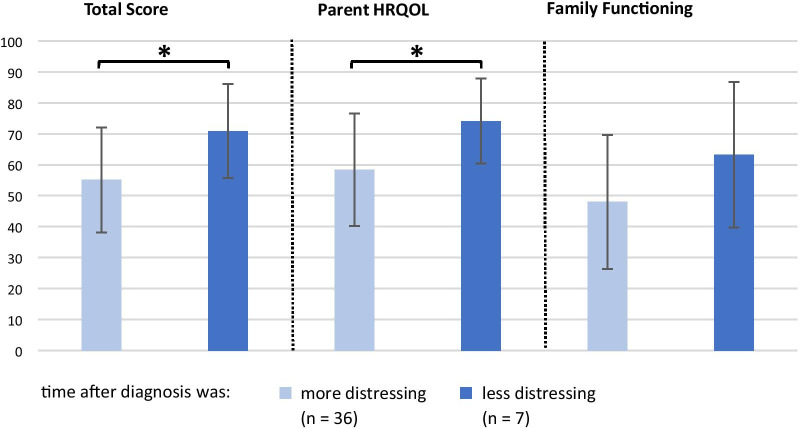
Influence of parents’ time after diagnosis on Quality of Life measures. Higher values equal better functioning/less negative impact. *P value is significant at < 0.05 level. Comparison between “more distressing” and “less distressing” samples. HRQOL, health-related quality of life

The parents were then divided into two groups according to the results on the above-mentioned rating scale: scores between 0 and 5 were classified as the “less distressing” group (n = 7, median time between diagnosis and survey: 8.5 years, range 4–7 years) and scores > 5 as the “more distressing” group (n = 36, median time between diagnosis and survey: 10 years, range 1–27 years). Comparing the means of these two groups in the PedsQL™ FIM revealed that the parents who rated the time following the diagnosis as more distressing had a significantly poorer functioning than the parents who did not. This was found in the overall domains of Total Impact Score (*P* < 0.05) and in Parent HRQOL Summary Score (*P* < 0.05) (Fig. 2).

### Influence of the severity of the symptoms on the family’s quality of life

#### Gross motor function and speech production

At the time of the survey, affected children (n = 45) had a median GMFC-MLD score of 5 (range 0–6), which equals “No locomotion nor sitting without support, but head control is possible” [16] and a median VSS score of 4 (range 1–4), which means “No understandable speech” [24]. Children with late-infantile MLD (n = 8) had a median GMFC-MLD score of 6 (range 5–6) and a median VSS score of 4 (range 1–4), children with juvenile MLD (n = 18) had a median GMFC-MLD score of 4.5 (range 0–6) and a median VSS score of 4 (range 1–4), and children with PCH2 (n = 19) had a median GMFC-MLD score of 5 (range 4–6) and a median VSS score of 4 (range 4–4). In summary, the groups did not differ substantially in their disease severity.

There was no significant correlation between GMFC-MLD or VSS scores and the family’s quality of life. In order to study the impact of gross motor function and speech production on the families’ quality of life, the families were divided into groups according to the abilities of their children, and the mean values from their PedsQL™ FIM were compared. Table 3 shows that there was no significant difference in HRQOL between families of children with a GMFC-MLD score of less than or equal to 4 and families of children with a GMFC-MLD score of above 4. Similarly, there was no significant difference between families of children with a VSS score of less than 4 and families of children with a VSS score equal to 4.

**Table 3:** Influence of severity of symptoms in PedsQL™ Family Impact Module

Sample	Total Impact Score	Parent HRQOL	Family Functioning
*Gross motor*			
GMFC-MLD ≤ 4 (n = 10/10/10)	61.7 (18.6)	63.3 (19.6)	59.3 (19.6)
GMFC-MLD > 4 (n = 32/32/31)	56.5 (17.4)	60.6 (18.2)	47.3 (22.7)
*Complete immobility*			
GMFC-MLD < 6 (n = 30/30/30)	61.8 (17.8)	64.2 (18.9)	56.0 (22.0)
GMFC-MLD = 6 (n = 12/12/11)	48.6* (13.5)	54.6 (15.8)	37.2* (18.3)
*Speech*			
VSS < 4 (n = 10/10/10)	62.5 (16.8)	65.0 (18.0)	60.5 (17.8)
VSS = 4 (n = 32/32/31)	56.6 (17.9)	60.3 (18.6)	47.9 (23.2)
Complete loss of communication			
VSS ≤ 4 (at least ability to express emotions) (n = 34/34/34)	61.5 (16.4)	65.1 (17.1)	54.3 (21.8)
No ability to express emotions (n = 8/8/7)	43.1** (15.3)	45.8** (15.9)	34.5 (19.3)
*Visual stimulation*			
Visual stimuli perceptible (n = 32/32/32)	62.2 (16.7)	65.8 (17.2)	54.1 (21.5)
Visual stimuli not perceptible (n = 10/10/9)	44.8** (13.9)	47.4** (15.0)	39.6 (23.4)
*Independent eating*			
Independent eating possible (n = 8/8/8)	66.3 (17.8)	67.7 (18.9)	64.5 (15.9)
No independent eating possible (n = 34/34/33)	56.1 (17.3)	60.0 (18.2)	47.7* (22.8)
*PEG tube*			
No PEG tube present (n = 23/23/23)	62.7 (15.7)	64.8 (17.9)	56.4 (18.6)
PEG tube present (n = 19/19/18)	52.3* (18.5)	57.3 (18.6)	44.0 (24.5)
*Final stage of disease*			
Child alive (n = 32/32/31)	61.7 (17.5)	64.7 (18.2)	55.4 (21.9)
Child deceased (n = 11/11/11)	44.5** (9.8)	49.3* (14.0)	34.2** (14.8)

Standard deviation presented in parentheses. Different case numbers result from the fact that one Family Functioning scale was insufficiently completed. Higher values equal better functioning/less negative impact

PedsQL™, Pediatric Quality of Life Inventory [23]; HRQOL, health-related quality of life; GMFC-MLD, Gross Motor Function Classification-MLD [16]; MLD, metachromatic leukodystrophy; VSS, The Viking Speech Scale [24]; PEG, percutaneous endoscopic gastrostomy

^*^P value is significant at < 0.05 level; **P value is significant at < 0.01 level. Comparison between every pair of a sub-item

#### Advanced disease stage

To study the impact of an advanced disease stage on the families’ quality of life, the families were divided into groups according to the severity of symptoms in their children and the mean values from their PedsQL™ FIM were compared (Table 3). Families with children who had a GMFC-MLD score of 6 (“loss of any locomotion as well as loss of any head and trunk control”) [16] had significantly poorer functioning than families of children with a GMFC-MLD score better than 6 in the overall domains of Total Impact Score and Family Functioning Summary Score. Furthermore, when families of children with a VSS score of 4 were divided into those where children still had the ability to express emotions and those where children could no longer express emotions, the latter had a significantly poorer functioning in the overall domains of Total Impact Score and Parent HRQOL Summary Score.

Other advanced symptoms also exhibited significant differences. Families of children who could no longer perceive visual stimuli or who could not eat independently, and who had a PEG (percutaneous endoscopic gastrostomy), as well as families whose children had already died at the time of the survey (and who reported on their situation during the end stage of the disease) had a significantly poorer functioning than the families of children without these advanced symptoms.

### Influence of the child’s illness on mothers and fathers

#### Comparison between mothers and fathers

**Fig. 3:**
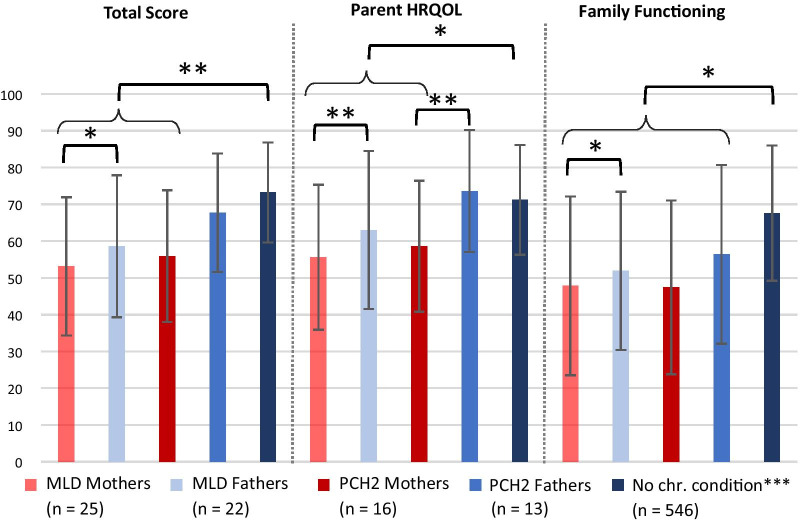
Comparison of HRQOL between mothers and fathers. Higher values equal better functioning/less negative impact. *P value is significant at < 0.05 level; **P value is significant at < 0.01 level. ***Medrano et al. [26]. Comparison between individual samples, as well as between individual samples and the ‘no chronic condition’ sample. MLD, metachromatic leukodystrophy. PCH2, pontocerebellar hypoplasia type 2. HRQOL, health-related quality of life. Chr., chronic

When investigating the influence of the child’s illness on mothers and fathers separately (mean scores see Table 2), the MLD mothers were found to have a significantly poorer functioning than the MLD fathers in the overall domains of Total Impact Score (*P* < 0.05), Parent HRQOL (*P* < 0.001) and Family Functioning (*P* < 0.05) Summary Scores (Fig. 3). The PCH2 mothers had a significantly poorer Parent HRQOL Summary Score (*P* < 0.001) than the PCH2 fathers.

In contrast, the comparisons between MLD and PCH2 mothers and between MLD and PCH2 fathers revealed no significant difference.

#### Professional situation of the parents

**Fig. 4:**
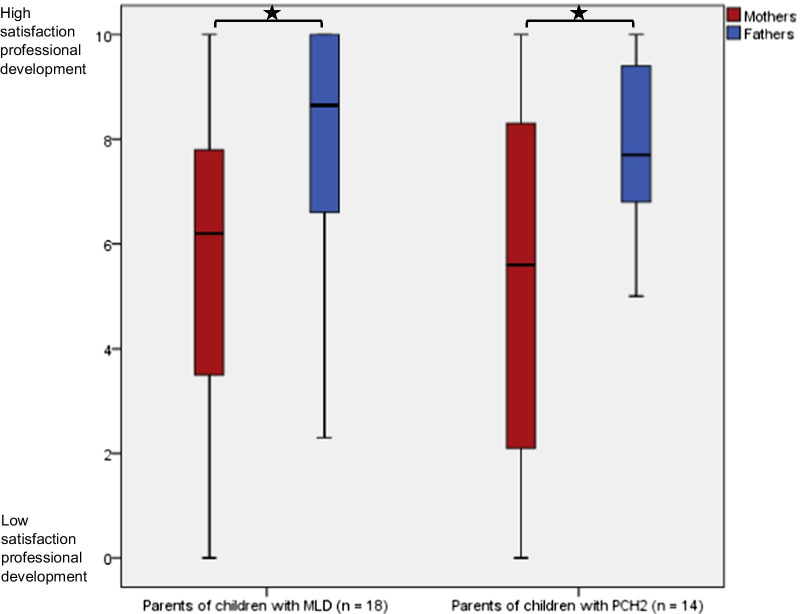
Satisfaction of parents with their professional development. Higher values equal better satisfaction with professional development. *P value is significant at < 0.05 level. Comparison between mothers and fathers. MLD, metachromatic leukodystrophy. PCH2, pontocerebellar hypoplasia type 2

Table 4 gives the educational level and current professional situation of parents (n = 43). It is noteworthy that that there seems to be no systematic difference in the degrees of education between the parents. At the time of the survey, 60.5% of mothers were working outside their homes and 25.6% were housewives, while 100% of the employed fathers worked outside their homes. Comparing the median values of a rating scale of 0 to 10, with 0 being “not satisfied with own professional development” and 10 “very satisfied with own professional development”, both mothers of children with MLD (n = 18, *P* < 0.05) and PCH2 (n = 14, *P* < 0.05) had significantly poorer satisfaction with their professional development than fathers (Fig. 4).

**Table 4:** Educational level and current professional situation in parents of affected children in the participating families (n = 43)

Variable	Mothers percent	Fathers percent
*Education*		
Certificate of secondary education	20.9	25.6
General certificate of secondary education	30.2	23.3
Advanced technical college entrance qualification	11.6	18.6
University-entrance diploma	32.6	27.9
Other	2.3	0.0
Not answered	2.3	4.7
*Current professional situation*		
Work outside home	60.5	76.7
Housewife/househusband	25.6	0.0
Retired	11.6	14.0
Deceased during lifetime of affected child	0.0	4.7
Not answered	2.3	4.7

MLD, metachromatic leukodystrophy; PCH2, pontocerebellar hypoplasia type 2

When asked whether their current professional situation corresponded to their original aims, 53.5% of the mothers and 9.3% of the fathers answered no and stated that their child’s illness played a significant role. For example, one parent commented,

“I had to quit my part-time job because I just could not make it anymore ([because of] bad nights, worries, [I] could not concentrate anymore and wanted to be with my child as well).” (late-infantile MLD)

### Influence of the child’s illness on non-affected siblings

Fifty percent of parents in MLD families with siblings (n = 24) reported that the experience of a seriously chronically ill sibling was a burden to the healthy child, whereas 16.7% rated it rather more positively. In PCH2 families (n = 11), 45.5% reported the experience of a seriously chronically ill sibling as rather more positive for the healthy sibling, whereas 9.1% rated it more as a burden. In free text, 20.8% of MLD parents and 18.2% of PCH2 parents indicated that this experience was both a burden, as well as positive, for the healthy child. A total of 12.5% of MLD parents and 27.3% of PCH2 parents didn’t answer this question.

When asked whether they had observed specific behavioral patterns in the healthy sibling which they thought were due to the illness of the affected child (multiple responses were possible), 57.1% of parents with healthy children (n = 35) specified that their healthy child “takes on a lot of responsibility in the family”, 46.4% that it “is often disadvantaged”, 42.9% that it “helps a lot with the care of the sick child” and 21.4% that it “shows behavioral problems like depression, aggression, retreat or hyperactivity”. Under the category “other”, 46.4% of parents reported on e.g. very good social skills in the healthy child but also on problems like a stutter or great insecurity. Twenty percent did not answer this question.

### Care and financial situation

At the time of the survey, 87.8% of the affected children (n = 49) had been allocated care by German institutions, 8.1% received no such allocation and 4.1% of parents did not answer this question. A total of 83.7% (n = 43) of those who had been allocated care were grade five, the highest level (“serious impairment of self-employment or abilities” [27]), meaning a very high requirement for care.

Mothers spent an average of 88 h and fathers of 39 h a week with their affected child. It was predominantly mothers who cared for the child in 60.5% of families (n = 43), both parents cared for the child equally in 20.9% of families, it was predominantly someone from outside the family, e.g. a nursing service, nursing home or boarding school, in 14.0% of families and predominantly fathers who cared for the child in 2.3% of families, while 2.3% did not answer this question.

A total of 69.8% of families stated they received help with the care of their child for an average of 38 h per week, whereas 20.9% stated they received no help, and 9.3% did not answer this question. When asked who helped with the care of the affected child (multiple responses were possible), 85.7% of families answered “friends”, 76.2% answered “relatives”, 54.8% answered “outpatient nursing services”, 69.0% answered “other”, e.g. “volunteers” or “hospice service”, and 2.3% did not answer this question. The reasons why some parents did not seek help were manifold. For example, one parent who didn’t receive any help wrote,

“I want to take care of my daughter myself for as long as I can.” (juvenile MLD)

And another, “I get help from relatives in the household. But they think that they’re not capable of caring for [name of the child].” (late-infantile MLD)

In this context, the influence of the child’s illness on the financial situation of the parents was also examined. On a rating scale of 0 to 10 with 0 being “no restrictions on our financial situation due to the illness of our child” and 10 “financially extreme restrictions due to the illness of our child”, the parents of MLD children (n = 27) specified a median value of 1.5 (range 0.0–10.0) and the parents of PCH2 children (n = 16) specified a median value of 5.0 (range 0.0–10.0).

### Social support for the family

Many parents stated that they mostly received comfort and encouragement from family and friends, to a minor degree from other affected families, and that family rehabilitation, health treatments and hospice stays were provided (Table 5). Under the category “other”, parents repeatedly stated that hobbies, activities and their work comforted them as they were a distraction.

**Table 5:** Sources of support for parents of affected children in the participating families (n = 43)

Source of support	Percent^a^
Family	73.8
Friends	66.7
Other affected families	28.6
Family rehabilitation/ health treatments /hospice	21.4
Other^b^	21.4
Religion	11.9
Patient support groups	9.5
Neighbors	2.4
Not answered	2.3

^a^Multiple responses were possible

^b^e.g. hobby, activities, work, therapist

On a rating scale of 0 to 10, with 0 being “I do not feel well accepted by society with my affected child” and 10 “I feel very well accepted by society with my affected child”, the parents of MLD children (n = 27) reported a median of 5.8 (range 2.5–10.0) and the parents of PCH2 children (n = 16) a median of 6.3 (range 2.2–10.0). A total of 76.7% of parents (n = 43) experienced “sympathy”, 69.8% “positive feedback” and 20.9% “lack of understanding” as public reactions to their affected child (multiple responses were possible). Under the category “other”, 25.6% of parents reported on a variety of public reactions to the affected child, such as interest and respect towards them, but also on insecurity and a sense of shame.

### Disease-specific knowledge in parents

On a rating scale of 0 to 10, with 0 being “I am currently not well informed about the illness of my child” and 10 “I am currently very well informed about the illness of my child”, the parents of MLD children (n = 27) reported a median of 8.4 (range 0.0–10.0) and the parents of PCH2 children (n = 16) a median of 9.0 (range 1.7–10.0). A total of 70.7% (n = 43) of parents indicated that they got their disease-specific knowledge from physicians, therapists and nursing staff, 51.2% from the exchange with other affected families, 46.3% from the Internet, 14.6% from specialist literature and 4.7% did not answer this question. Under the category “other”, 4.9% of parents reported that they obtained information from e.g. studies carried out by the University Hospital Tübingen or a doctor they had befriended (multiple responses were possible).

When asked what would be necessary to make parents even better informed about their child’s illness, one parent wrote,

“Maybe an even better nationwide exchange between doctors treating rare diseases, since for most [doctors] it is the first time they are treating the disease.” (PCH2).

And another parent noted, “One is so concerned with the care of the child that I wish the doctors would address you actively and communicate new information and insights.” (juvenile MLD).

## Discussion

In this study, the impact of severe rare chronic neurological disease in childhood on the quality of family life was investigated in a large group of families in Germany with children suffering from MLD and PCH2. A common element to both diseases is that they are rare and severe childhood neurological disorders, with the difference that PCH2 is associated with a primary severe developmental disorder, while children with MLD initially develop normally and then progressively deteriorate [10, 13].

We found that the child’s illness had a significant negative influence on the quality of family lives. Interestingly, comparing the two diseases did not reveal major differences. It is of note here that, at the time of the survey, the majority of the affected children exhibited a similarly high requirement for care, with a median GMFC-MLD score of 5 and a median VSS score of 4, indicating the loss of almost all gross motor and communication skills. Irrespective of the disease, mothers had a significantly lower HRQOL and were significantly more dissatisfied with their professional development than fathers. Time to diagnosis, in particular, which was usually very long, meant a heavy burden on the parents, while they were generally quite satisfied with the way the diagnosis was communicated. Severe symptoms in the terminal stages of disease seem to be the other very distressing period associated with the disease based on the reported functioning for families during that stage.

### Negative impact of MLD and PCH2 on HRQOL of families

Whereas a poorer HRQOL than in families with healthy children has already been reported in MLD patients [3, 28], the impact of PCH2 on parents and the family has not yet been shown. We report that both the Parent HRQOL and Family Functioning are significantly lower in MLD and PCH2 families compared to published reference data from families with healthy children [26].

### Poorer HRQOL and satisfaction with professional development in mothers

A new finding of our study is the marked differences between mothers and fathers. MLD mothers had significantly poorer functioning than MLD fathers and PCH2 mothers had significantly poorer Parent HRQOL than PCH2 fathers. Most studies only examine the influence of a child's illness on the mother and little is therefore known about the effect on fathers. A previous study of families coping with cystic fibrosis reported that a lack of togetherness potentiated maternal distress. Fathers were often not available due to professional obligations or felt that caring for children was simply a woman’s work [29]. Moreover, in this study, mothers of both children with MLD and PCH2 were significantly less content with their professional development than fathers, which could be another possible reason for their poorer functioning. Looking for reasons for this significantly poorer contentment, half of the mothers, but less than 10% of fathers, stated that their current professional situation did not correspond to their original aims and this was seen as related to their child's disease. It is interesting to note in this context that the level of education was similar in mothers and fathers. Nearly all parents at least had a certificate of secondary education and a third of mothers and slightly fewer fathers had a University-entrance diploma. However, only 60% of mothers versus 77% of the fathers were working outside their homes. In addition, it was predominantly the mother who cared for the child in 60% of families versus both parents in equal parts in around 20% of families, and predominantly fathers in only 2% of families. These results show that, in many families, mothers either leave professional fulfilment behind in favor of the care of their sick child or suffer from the double burden of care and work. In a study on mothers of children with type 1 diabetes, many reported that they found it necessary to reduce their working hours or even change their workplace because of their child’s chronic illness, although work was actually a source of enrichment for these mothers [30]. In addition to the requirement for care, mothers in our study repeatedly reported the great desire to take care of their sick child themselves and thereby accepted the traditional role, but simultaneously suffered due to the fact that the care of their seriously ill child made a professional career difficult. With respect to fathers, it is of note that PCH2 fathers seemed relatively less affected, as they scored close to families with healthy children with respect to Total Impact Score and Parent HRQOL Score, with only the score on Family Functioning being significantly lower. The sample size of 13 PCH2 fathers is rather small, and a certain selection bias cannot be excluded. Alternatively, a primary severe developmental disorder, such as PCH2, may allow fathers to cope better with the challenge of combining family life with a child needing much care with career development than MLD fathers, whose children develop normally to begin with and then suddenly deteriorate progressively.

Although neither the dynamics of the disease, primary versus secondary progressive, nor the severity of symptoms, made a major difference with respect to the reduced parent and family functioning, two disease stages were clearly reported as especially distressing and burdensome.

### Time to diagnosis represents a high burden

The period when the diagnosis was not yet known was usually very long, on average 25 months from the onset of first symptoms. Many reported an odyssey, with many visits to different medical facilities. Unfortunately, diagnostic delays are common in rare diseases and have been documented before for MLD [3, 5, 17]. Needless to say, the worries of parents about their child’s development have to be considered carefully by the physician. Experiences reported by the parents here document the need to address the challenge with rare diseases in educational programs. Nevertheless, there were also parents who retrospectively rated this period to diagnosis as not distressing. A common reason for this seemed to be that they were not alarmed by the symptoms of their child and simply did not expect a bad diagnosis.

### Negative impact of advanced symptoms on HRQOL of families

A very advanced or terminal stage of their child’s disease was the other stage reported as specifically distressing and had a significant negative impact on the functioning of families. Advanced symptoms were complete immobility, complete loss of communication, blindness, loss of independent eating or the requirement for a PEG tube. In more detail, complete loss of communication and blindness primarily affected Parent HRQOL, while complete immobility and the loss of independent eating had more of an effect on Family Functioning. Previous reports on children with life-limiting conditions other than MLD or PCH2 have shown that these severe impairments are difficult to manage and significantly impact the quality of life of families with children with degenerative brain diseases [1]. Especially symptoms which indicate progression of the disease are known to be a burden on families [2].

While the diagnostic delay discussed above constitutes rather problematic feed-back for our health care system, parents’ fairly positive responses on how the diagnosis was communicated is more reassuring. Feedback is that doctors generally informed parents of the diagnosis in their offices. Honesty about the severity, a comprehensible explanation of the condition, as well as empathic handling were all reported as very helpful. On the other hand, insensitive behavior towards parents, ignorance in physicians about the disease and its clinical implications, as well as unconfirmed or even incorrect diagnoses, were all perceived as very distressing. The need to treat families respectfully and supportively, as well as to provide them with clear and up-to-date information has been emphasized in previous studies on family-centered support [6, 31].

Although, the time after diagnosis was experienced as very distressing by many parents, some also reported that, in a way, they felt relieved by the diagnosis as it provided them with the opportunity to learn about the disease and contact other families affected by the same disease. Another important point for these parents was the diagnosis of MLD or PCH2 as hereditary diseases, which explained the origin and showed that they had no responsibility for the illness of their child. These parents also had a significantly better functioning than parents who rated the time after diagnosis as very distressing. This indicates that impact on parents with a pragmatic attitude and good medical knowledge is less negative. This is in line with studies on family-centered support, showing that a category of active parents want information and resources in order to be effective decision-makers [6].

### Good disease-specific knowledge in parents

Immediately after diagnosis, parents of MLD children felt neither well informed, nor insufficiently informed about their child’s disease, whereas parents of PCH2 children initially felt rather more badly informed. This could be an indicator that pediatric neurologists in Germany know less about PCH2 than about MLD. However, it is reassuring that parents of both MLD and PCH2 children currently felt well-informed about their child’s disease. This is in line with what has been argued, that family-centered support should include providing parents with up-to-date information to encourage them to make decisions for their families and thus play an active role in the care of their child [6, 31, 32]. Parents mainly obtained information from doctors, therapists or nursing staff, which highlights the important role that health care professionals play for affected families. In addition, exchanges with other affected families and the Internet were used to obtain information. In turn, parents wished that doctors treating children with rare diseases would engage more in these diseases, as they had often experienced a lack of knowledge in physicians and other health care professionals with respect to their child’s disease.

### Different effects of the child’s illness on unaffected siblings

An important observation of this study was that parents rated the experience of a seriously, chronically ill sibling on the unaffected sibling in very different ways. While MLD parents were more likely to find that the severe disease in the affected sibling was more of a burden to the healthy child, PCH2 parents rated this experience as more positive. Similar to our results, a literature review on siblings of children with cancer and autism also revealed positive as well as negative reactions in the healthy siblings [22]. In our study, around 20% of MLD and PCH2 parents even rated the experience for the healthy child as both a burden and a positive experience, although this option was not available in the general questionnaire. Parents described their healthy children, as very responsible and helpful and with very good social skills, but also reported that they often become disadvantaged and may show behavioral problems such as depression, aggression or hyperactivity. It is therefore essential to be aware that living with MLD or PCH2 children may have harmful effects on some siblings, so that health professionals can also take supportive measures to protect the well-being of siblings.

### Social support sources for parents

A positive finding of our study was that most parents felt socially accepted with their affected child. More than two thirds answered that they gain comfort and encouragement in their own environment from family and friends and almost a third considered the exchange with other affected families as helpful. Only about 20% of families received comfort and encouragement from professional services such as family rehabilitation, health treatments or hospice stays. Some parents indicated they gained comfort from hobbies, activities and their work, as it distracts them. Regarding care and nursing of the children, most parents again primarily received help in their own environment from family and friends, more than half were also supported by outpatient nursing or hospice services. However, around 20% of families indicated they did not receive any help. Previous studies have found that family-centered professional support was positively associated with family quality of life [8, 31–33] and our data may indicate that the low use/availability of professional services could be one possible reason for poor functioning of families with children with MLD or PCH2. Health care professionals should therefore be aware of the importance of delivering supportive services to families of children with MLD or PCH2.

### Limitations

Of course, a study like this that addresses rare diseases such as MLD or PCH2 has clear limits, starting with the number of participating parents, the incomplete answering of individual questions, and a potential for bias based on those who have agreed to participate in this study. Parents of children suffering from MLD and PCH2 were known to the authors from previous studies and had been recruited via the nationwide Leukonet research association, patient support groups and the University Hospital Tübingen, and were asked to participate; selection was therefore based on the agreement to participate and, thus, not randomized.

Part of the general questionnaire recording the current stage of disease was based on previously validated and established tools, such as the GMFC-MLD [16] and the VSS [24]. The GMFC-MLD should be applied from the age of 18 months onwards, with the reference being the ability to walk. The VSS was designed for and tested with children aged four and above. We also used them in younger children for comparability of functional states defined in these scales.

We did not test the PedsQL™ Family Impact Module [23] in families with normally developing children, but used the published reference data from a large cohort of families with healthy children from the USA [23]. Although no major differences of parents’ health-related quality of life and family functioning are expected, the different country and culture of the reference group might influence comparability.

At the same time, the scope of the general questionnaire had to be limited in order to maintain the motivation of the participants. Consisting of eight pages of data collection and the PedsQL™ FIM containing 36 items, the questionnaire tool had become rather extensive. Use of a shortened form of the instrument should therefore be considered in follow-up examinations. The possibility for open text and comments provided qualitative data, proved to be very informative and should by all means be retained.

## Conclusions

- Severe, rare chronic neurological disorders in childhood, studied here using the examples of MLD and PCH2, are a heavy burden on the entire family system and the life perspectives of the family members.
- Mothers proved to be particularly affected. More often than fathers, they missed out on or sacrificed a professional career in order to be able to care for the child at home.
- Healthy siblings reacted in different ways. Even though they sometimes showed positive behavioral changes, it should be borne in mind that living with a sibling affected by MLD or PCH2 is a burden on the healthy brother or sister as well and they also need attention and supportive measures to protect their well-being.
- Time to diagnosis proved to be long and distressing for parents, which highlights the importance of further increasing a general awareness, that although rare diseases are by definition rare, taken together their number is high and origin increasingly discovered. Medical professionals in their daily routine are likely to encounter such a disease. MLD and PCH2 are but examples of such diseases and the experience reported here shows how important it is to listen to parents who report that they are worried about developmental and behavioral features of their children, they have not known before.
- A reliable system of easily accessible diagnostic counseling for physicians and families, which should involve academic centers and define their responsibility for promoting rapid diagnosis of rare diseases is needed.
- Furthermore, the terminal disease stage represents an especially high burden on families. The heavy demands on care, on the one hand, and minimal feedback from the child at that stage, on the other, are possible reasons for this. Not all families have taken advantage of professional support yet, although we know that providing comprehensible and up-to-date information, as well as individually tailored professional help, has a positive effect on families. Parents and families of children with MLD and PCH2 would benefit from family-centered professional support due to the multiple challenges of living with a child with a severe rare chronic neurological disorder.

## Data Availability

The datasets generated and analyzed during the current study are not publicly available due to protection of the privacy of the individuals, but may be made available anonymized upon request addressed to the corresponding author, pending the approval of the Institutional Review Board of the University of Tuebingen, Germany.

## Abbreviations

ADHD: Attention Deficit Hyperactivity Disorder
ASA: Arylsulfatase A
Chr.: Chronic
FIM: Family Impact Module [23]
GMFC-MLD: Gross Motor Function Classification-MLD [16]
HRQOL: Health-related quality of life
MLD: Metachromatic leukodystrophy
PCH: Pontocerebellar hypoplasias
PCH2: Pontocerebellar hypoplasia type 2
PedsQL™: Pediatric Quality of Life Inventory [23]
PEG: Percutaneous endoscopic gastrostomy
SD: Standard deviation
VSS: The Viking Speech Scale [24]
